# Supplementary material for: The shield of self-compassion: A buffer against disordered eating risk from physical appearance perfectionism
Source: PLoS One. 2020 Jan 13;15(1):e0227564. doi: 10.1371/journal.pone.0227564 (PMC6957174; doi:10.1371/journal.pone.0227564)
Supplement: S1 Table — This includes mean (M), standard deviation (SD) and range (N = 421). (PDF) [file pone.0227564.s001.pdf]

S1 Table: Summary of descriptive statistics for untransformed and transformed values. This includes mean (M), standard deviation (SD) and range (N=421).

	Raw values			Transformed values		
**Variable**	*M*	*SD*	*Range*	*M*	*SD*	*Range*
1. Self-oriented perfectionism (MPS)	26.80	6.31	30	3.73	1.06	4.57
2. Disordered Eating Symptoms (EAT-26))	11.45	11.74	54	2.93	1.70	7.35
3. Body Mass Index (BMI)	22.24	3.20	18.33	1.34	.06	.33

**Variable**	R^2	ΔR^2	B	SE(B)	β	p
Step 1	.34	.34				
Constant			-12.65 (-16.36, -9.17)	1.86		<.001
Worries about appearance imperfection			5.44 (4.38, 6.48)	.49	.50	<.001
Striving for appearance perfection			1.94 (.87, 3.00)	.52	.17	.001
Step 2	.36	.02				
Constant			-17.94 (-22.92, -13.55)	2.35		<.001
Worries about appearance imperfection			5.17 (4.12, 6.20)	.50	.47	<.001
Striving for appearance perfection			1.52 (.44, 2.58)	.52	.13	.010
Socially-prescribed perfectionism			.08 (-.08, .24)	.08	.04	.320
Self-oriented perfectionism			.23 (.07, .39)	.08	.12	.007

Note: R^2= r_squared. ΔR^2=change in R^2. B= unstandardized regression coefficient. SE(B)= standard error B. β = standardised regression coefficient.

**Variable**	R^2	ΔR^2	B	SE(B)	β	p
Step 1	.23	.23				
Constant			2.68 (1.01, .52)	.94		.004
DASS21			.43 (.33, .52)	.04	.48	<.001
Step 2	.26	.03				
Constant			-5.54 (-9.46, -1.66)	2.21		.013
DASS21			.37 (.28, .46)	.04	.42	<.001
Socially prescribed perfectionism			.10 (-.07, .28)	.09	.06	.229
Self-oriented perfectionism			.28 (.12, .45)	.09	.15	.002
Step 3	.41	.15				
Constant			-16.77 (-21.57, -12.52)	2.29		<.001
DASS21			.22 (.13, .31)	.04	.25	<.001
Socially prescribed perfectionism			-.01 (-.18, .14)	.08	-.01	.855
Self-oriented perfectionism			.19 (.04, .35)	.08	.10	.020
Worries about appearance imperfection			3.99 (3.01, 4.95)	.53	.36	<.001
Striving for appearance perfection			1.76 (.74, 2.87)	.51	.15	.001

Note: R^2= r_squared. ΔR^2=change in R^2. B= unstandardized regression coefficient. SE(B)= standard error B. β = standardised regression coefficient.
