# Supplementary material for: The shield of self-compassion: A buffer against disordered eating risk from physical appearance perfectionism
Source: PLoS One. 2020 Jan 13;15(1):e0227564. doi: 10.1371/journal.pone.0227564 (PMC6957174; doi:10.1371/journal.pone.0227564)
Supplement: Attachment — Submitted filename: Review_Oct17.docx [file pone.0227564.s002.docx]

The shield of self-compassion: a buffer against disordered eating risk from physical appearance perfectionism

Thank you for the opportunity to review this manuscript. The authors conducted an interesting study exploring the dimensionality of perfectionism as it relates to self-compassion, and modelled both in the context of disordered eating in a sample of female university students.

Background and introduction:

1. Literature review for perfectionism is, at times, confusing. From the reviewed articles in the introduction, the consensus appears that various assessments tap into varying dimensions of perfectionism. It is unclear which dimensions are of particular interest to the authors. These sections could benefit from some reorganization to highlight either the existing diversity in the understanding of perfectionism dimensionality, or if the literature is well-established in select dimensions, have a more focused discussion surrounding those specific dimensions of interest.
2. I wonder if the authors could clarify the proposed model and role of self-compassion a little more. In text, the model appears as one of moderation. The graphical representation of this model via Fig. 1 appears more like divergent trajectories marked by adaptive vs. maladaptive coping strategies in response to levels of self-compassion (mediated moderation). Perhaps both in-text description and graphical representation could be better harmonized.
3. Further in response to the discussion of adaptive vs. maladaptive coping strategies (in Fig 1), the emotion regulation literature has also suggested that psychological flexibility is more adaptive overall as strategies like reappraisal may not always be used “adaptively”. For example, some may reappraise the situation to be much more negative than it is. In addition, previously regarded negative coping, such as avoidance, has some basis from a functional contextualism view. Self-compassion may be more closely tied to psychological flexibility, which speaks to the underlying mechanisms of self-compassion, including acceptance, mindful awareness, and non-judgement (DOI: 10.1891/0889-8391.30.1.60).
4. Lines 149-150. With changes in self-perception in the age of social media, a challenge may be that the manifestations of DES is not well conceptualized in males. I suggest the authors rephrase this sentence to the tune of… “Given challenges in the conceptualizations and manifestations of DES in males, we limited the recruitment of only female university students…”.
5. Lines 159-159. Since the authors only recruited females in their study, the statement in relation to women does not apply. Suggest to reword.

Methods:

1. Aside from the possibility of receiving a gift card, where participants otherwise compensated for their time? If not, please discuss limitations of opportunity sampling in relation to self-selection bias in the discussion section.
2. Please include reliability statistics of the sampled population (Cronbach’s alpha) for each measure used.
3. 25% missing data is a significant amount to be replaced with mean. Were the mean replacement values the sample mean or individual mean? Were the missing data missing at random, or more concentrated to specific measures?
4. Given the sensitivity of modelling new relationships and the large quantity of missing data, I suggest the authors use an alternative method to deal with missing values that accounts for more error biases in data (e.g., see <https://www.ncbi.nlm.nih.gov/pmc/articles/PMC3668100/>)
5. What data transformation was used? Would be helpful if authors produced a table (supplementary is also okay) documenting the raw means, sd, range and the transformed values.

Results:

1. Please note that the DASS-21 is a self-report measure of depression, anxiety, and stress, and should be reported as three separate subscales rather than collapsed into a single variable (Lovibond, S.H. & Lovibond, P.F. (1995). Manual for the Depression Anxiety & Stress Scales. (2nd Ed.)Sydney: Psychology Foundation.). Subsequent analyses should adopt the three-subscale variable structure, and re-analyzed.
2. The rationale for why general perfectionism was entered in step 2 of the hierarchical regression (rather than step 1) was not clearly articulated.
3. Would the authors please include a table of model coefficients for all variables in the hierarchical regressions conducted?
4. Did the authors account for the shared variance of these variables prior to the regression through a single block entry? If not, what was the justification for the order of entry given that correlations at least suggest there may be some overlap or shared variance?
5. Line 228 indicated PROCESS macro would be used for the purpose of conducting moderation. Why did the authors choose a hierarchical regression in lieu of PROCESS?

Discussion:

1. A central theme of this paper is the author’s modification of the PAPS from “hope” to “strive”. Could the authors elaborate more on why a change in terminology may have tapped into a different dimension that the previous version was not successful in tapping into?
2. Can the authors speak more to why there was an interaction with worries-about-appearance imperfection when those in low perfectionism and high in self-compassion also were showed highest in disordered eating? What are some of the potential confounds not otherwise controlled for? For example, if it is the existence of high distress, the “stress” subscale of the DASS-21 would at least tap into the self-reported aspect of it (and similarly for self-reported levels of depression and anxiety).
3. Given the interaction that did not support the hypothesized role of self-compassion as a moderator of perfectionism and DES, the extent to which results support self-compassion as a moderator should be more cautiously interpreted.
4. It would be important for the authors to discuss some cultural implications and variations in self-compassion as it relates to disordered eating and coping, as well as discuss how results may or may not be different in a male sample, older/younger samples, etc.

Minor:

- There are several instances where the paragraph breakdown appears misaligned. This is likely an issue when adapting word format to pdf, or vice versa. May be worth fixing for ease of review.
