# Supplementary material for: The shield of self-compassion: A buffer against disordered eating risk from physical appearance perfectionism
Source: PLoS One. 2020 Jan 13;15(1):e0227564. doi: 10.1371/journal.pone.0227564 (PMC6957174; doi:10.1371/journal.pone.0227564)
Supplement: Attachment — Submitted filename: Response to Reviewers PLOS one.docx [file pone.0227564.s003.docx]

Dear Dr. Vickers,

Thank you for identifying areas in need of improvement in our paper titled “The shield of self-compassion: a buffer against disordered eating risk from physical appearance perfectionism” and thereby giving us the opportunity to strengthen our research prior to publication. We appreciate the time and effort you and the reviewers have dedicated to delivering detailed feedback on our manuscript. We have been able to incorporate changes to reflect most of the suggestions provided by the reviewers.

Here is a point-by-point response to the reviewers’ comments and concerns.

**Data Availability**

**Required Revision 1:** *It is extremely important that* ***the data be available****. Despite using different computers and browsers, I was not able to open the data using the link provided. Attempts to do so resulted in an error message saying: DOI Not Found  10.17630/39426414-eb9b-4b7f-b078-2f88f96cdd76  Reviewer 2 also noted this issue.*

**Response**: Thank you for pointing this out. We have been in contact with the University of St Andrews Research Data and Information Services. The DOI (<https://doi.org/10.17630/39426414-eb9b-4b7f-b078-2f88f96cdd76>) has now been activated and the data supporting our research can be found here.

**Introduction**

**Reviewer #1**

**Comment 1**: *Negative Affect (NA) is sometimes presented as a control variable, sometimes as a moderator and sometimes as a predictor. In my experience, it’s customary to make one such prediction rather than casting one variable in all three roles. See the following instances, with line numbers embedded*

**Response**: Thank you for pointing this out. In the hierarchical regressions we included negative affect as a predictor in order to assess the effects of general and physical appearance perfectionism above this known predictor of disordered eating symptoms. Negative affect was used as a control variable in the moderation analysis as prior research suggests it predicts variance both in perfectionism levels and disordered eating symptoms. We have made this distinction clearer in the introduction and results.

**Reviewer #2**

**Comment 1:** *Literature review for perfectionism is, at times, confusing. From the reviewed articles in the introduction, the consensus appears that various assessments tap into varying dimensions of perfectionism. It is unclear which dimensions are of particular interest to the authors. These sections could benefit from some reorganization to highlight either the existing diversity in the understanding of perfectionism dimensionality, or if the literature is well-established in select dimensions, have a more focused discussion surrounding those specific dimensions of interest.*

**Response**: Thank you for highlighting this. We have extended our discussion of the dimensions of perfectionism which are of particular interest in our study and have given more detailed definitions of the respective dimensions in our introduction. As we are examining general perfectionism and physical appearance perfectionism we focus on these constructs. We have expanded our discussion of general perfectionism to indicate why we are focussing on its subcomponents of striving and concern.

**Comment 2**: *I wonder if the authors could clarify the proposed model and role of self-compassion a little more. In text, the model appears as one of moderation. The graphical representation of this model via Fig. 1 appears more like divergent trajectories marked by adaptive vs. maladaptive coping strategies in response to levels of self-compassion (mediated moderation). Perhaps both in-text description and graphical representation could be better harmonized.*

**Response**: Thank you for this helpful comment. We have addressed it by changing the wording in figure 1. The mediating role of emotion regulation is simply a suggestion for a mechanism as to how or why self-compassion may exert its protective effect. However, in our study we were not testing this and instead concentrating on the role of self-compassion as moderator.

**Comment 3**: *Further in response to the discussion of adaptive vs. maladaptive coping strategies (in Fig 1), the emotion regulation literature has also suggested that psychological flexibility is more adaptive overall as strategies like reappraisal may not always be used “adaptively”. For example, some may reappraise the situation to be much more negative than it is. In addition, previously regarded negative coping, such as avoidance, has some basis from a functional contextualism view. Self-compassion may be more closely tied to psychological flexibility, which speaks to the underlying mechanisms of self-compassion, including acceptance, mindful awareness, and non-judgement (DOI: 10.1891/0889-8391.30.1.60).*

**Response**: Thank you for raising this interesting point. We have included a discussion of the role of psychological flexibility in our introduction and have refrained from labelling emotion regulation strategies as generally adaptive or maladaptive and instead focussed on situation-appropriate strategy selection and use. It is important to point out that although research has found self-compassion and psychological flexibility to be related, self-compassion was found to explain variance in well-being beyond psychological flexibility (Marshall & Brockmann, 2016; *DOI: 10.1891/0889-8391.30.1.60*).

Comment 4: *Lines 149-150. With changes in self-perception in the age of social media, a challenge may be that the manifestations of DES is not well conceptualized in males. I suggest the authors rephrase this sentence to the tune of… “Given challenges in the conceptualizations and manifestations of DES in males, we limited the recruitment of only female university students…”.*

**Response**: Thank you for this helpful comment. We have rephrased the sentence to better justify our choice of sample and have added a recent review paper as a reference, which highlights current lack of valid assessment tools for disordered eating in males.

**Comment 5**: *Lines 159-159. Since the authors only recruited females in their study, the statement in relation to women does not apply. Suggest to reword.*

**Response**: Thank you. We agree and have reworded the sentence to reflect this comment.

**Methods**

**Required Revision 1:** *Please report the reliability values for the measures as reflected in the current sample (as noted by Reviewer 2).*

**Comment Reviewer #2**: *Please include reliability statistics of the sampled population (Cronbach’s alpha) for each measure used.*

**Response**: Thank you for this suggestion and highlighting the importance of internal reliability for measures used. However, we would like to point out that Cronbach’s Alpha for each measure used in the current sample is reported in our manuscript in the final column of Table 1.

**Required Revision 2:** *Please present the DASS-21 as 3 subscales (as noted by Reviewer 2).*

**Comment Reviewer #2:** *Please note that the DASS-21 is a self-report measure of depression, anxiety, and stress, and should be reported as three separate subscales rather than collapsed into a single variable (Lovibond, S.H. & Lovibond, P.F. (1995). Manual for the Depression Anxiety & Stress Scales. (2nd Ed.)Sydney: Psychology Foundation.). Subsequent analyses should adopt the three-subscale variable structure, and re-analyzed.*

**Response**: You have raised an important point here. However, based on recent research findings from Osman et al. (2012) -  <https://doi-org.wwwdb.dbod.de/10.1002/jclp.21908> - we argue that it is more appropriate to represent the DASS21 as a total score of negative affect or general distress. Osman et al. (2012) investigated the factor structure of the DASS21 in a large sample of nonclinical US university students and found strong evidence for a general distress factor which accounted for the majority of the variance in the DASS21 individual items and the total score. They conclude that the DASS21 is “a theoretically relevant measure of negative emotions that include mixed symptoms of depression, anxiety, and stress”. To enhance our manuscript, we have included this reference and an explanation for using the DASS21 as a measure of negative affect in the methods section of our manuscript.

**Required Revision 3:** *Please describe the transformations applied and how the transformations were selected (as noted by both reviewers).*

**Comment Reviewer #1**: You briefly refer to data transformations: give specifics.

**Comment Reviewer #2**: What data transformation was used? Would be helpful if authors produced a table (supplementary is also okay) documenting the raw means, sd, range and the transformed values.

**Response**: We agree and have included a more detailed description of the transformations applied. Following Reviewer 2’s suggestion, we have included a table documenting the raw means, sd, range and transformed values for the respective variables (please see supplementary materials for the table).

**Reviewer #2**

**Comment 1:** *Aside from the possibility of receiving a gift card, where participants otherwise compensated for their time? If not, please discuss limitations of opportunity sampling in relation to self-selection bias in the discussion section.*

**Response**: Thank you for this comment. Aside from the possibility of receiving the gift card, participants were not otherwise compensated for their time. Hence, we have now included a discussion of the limitations associated with opportunity sampling.

**Results**

**Required Revision 1:** *Please clarify whether the authors are referring to the analysis performed by the PROCESS macro (for moderation) as regression. (Reviewer 2 also notes this issue, Results comment 5; it appears that the reviewer thought that the PROCESS macros was not used, given the way in which the analyses were described). Did the authors use the PROCESS macro? If so, please clarify the language. As Hays notes (*[*http://processmacro.org/faq.html*](http://processmacro.org/faq.html)*) the regression approach may yield the same results as PROCESS but differences are also possible.: “…When the same model is estimated using the same data with the same output options, the results will be the same as what you get with SPSS or SAS's regression procedures.  There are many sources of discrepancies you may notice when discrepancies exist...A common one is requesting heteroscedastity-consistent standard errors in PROCESS, which are different than standard OLS standard errors.  SPSS and SAS won't generate these standard errors, but PROCESS will (as will my RLM and HCREG macros) but only if you ask for them.  When you do, standard errors, t-values, p-values, and confidence intervals are different than what SPSS and SAS's internal regression procedures produce, as they should be.”*

**Comment Reviewer #2**: *Line 228 indicated PROCESS macro would be used for the purpose of conducting moderation. Why did the authors choose a hierarchical regression in lieu of PROCESS?*

**Response**: Thank you for highlighting this very important point. We would like to clarify that PROCESS was used in the moderation analyses. We have adjusted our description of the analyses in the results section to reflect this. We have further used heteroscedasticity-consistent standard errors and adjusted the affected results.

Reviewer #2

**Comment 1**: *The rationale for why general perfectionism was entered in step 2 of the hierarchical regression (rather than step 1) was not clearly articulated.*

**Response**: Thank you for pointing this out. Please see our response to comment 3 below for a detailed explanation. We have more clearly explained the rationale for this choice in our manuscript introduction.

**Comment 2**: *Would the authors please include a table of model coefficients for all variables in the hierarchical regressions conducted?*

**Response**: Thank you for your comment. We have included a table of model coefficients for all variables in the two hierarchical regressions conducted.

**Comment 3**: *Did the authors account for the shared variance of these variables prior to the regression through a single block entry? If not, what was the justification for the order of entry given that correlations at least suggest there may be some overlap or shared variance?*

**Response**: Thank you. We did not use a single block entry to account for the shared variance prior to the regression due to the fact that we had a theoretical rationale for entering our variables in the order that we did. The single block entry method is often considered controversial (Tabachnik & Fidell, 2007) and mainly useful in exploratory research. The rationale for entering physical appearance perfectionism first and then general perfectionism was to assess the unique amount of variance accounted for in disordered eating symptoms by general perfectionism above that accounted for by physical appearance perfectionism. In the second hierarchical regression, negative affect was entered first to assess the variance uniquely accounted for by this known risk factor. Then general perfectionism was entered to again assess the unique variance accounted for in disordered eating above that accounted for by negative affect. Finally, physical appearance perfectionism was entered, our main variable of interest in this case, to accurately assess the unique additional variance explained in disordered eating by physical appearance perfectionism. Where possible, we have attempted to make this rationale clearer in our manuscript in the introduction.

**Discussion**

**Reviewer #1**

**Comment 1:** *The weight distribution does not seem to match population stats. Probably because it is self-reported. This idea in the discussion, but only briefly. Please enhance discussion. The way it is currently presented it seems like you are saying BMI doesn’t predict correctly when it really may have been measurement error. See quote:“BMI 240 was unrelated to all variables and hence not included as a control variable subsequently.”*

**Response**: Thank you for pointing this out. We have addressed this both in the results and the discussion by explaining in more detail that this finding was unexpected and that it may be due to measurement error.

**Reviewer #2:**

**Comment 1:** *A central theme of this paper is the author’s modification of the PAPS from “hope” to “strive”. Could the authors* ***elaborate*** *more on why a change in terminology may have tapped into a different dimension that the previous version was not successful in tapping into?*

**Response**: Thank you for your comment. We have included a more detailed description of the rationale for the modification in the introduction as well as an additional sentence in the discussion.

**Comment 2:** *Can the authors speak more to why there was an interaction with worries-about-appearance imperfection when those in low perfectionism and high in self-compassion also were showed highest in disordered eating? What are some of the potential confounds not otherwise controlled for? For example, if it is the existence of high distress, the “stress” subscale of the DASS-21 would at least tap into the self-reported aspect of it (and similarly for self-reported levels of depression and anxiety).*

**Response**: Thank you for your comment. We have included your advice by elaborating in the discussion session on potential variables which may explain the crossover interaction but were not included in our study. We already controlled for negative affect in our moderation analyses. We only controlled for negative affect as one variable based on the findings from Osman et al (2012), which suggest that a general distress factor explains more variance in DASS-21 than the separate subscales of depression, anxiety and stress.

**Comment 3:** *Given the interaction that did not support the hypothesized role of self-compassion as a moderator of perfectionism and DES, the extent to which results support self-compassion as a moderator should be more cautiously interpreted.*

**Response**: Thank you. We see your point and added a cautioning statement in our limitations section. However, considering the large sample and the use of robust methods, we do believe it is valuable to highlight that the findings suggest self-compassion may function as a moderator.

**Comment 4:** *It would be important for the authors to discuss some cultural implications and variations in self-compassion as it relates to disordered eating and coping, as well as discuss how results may or may not be different in a male sample, older/younger samples, etc.*

**Response**: We agree. To address this comment, we have included a more detailed discussion of our sample in the limitations section of the discussion.

We look forward to hearing from you in due time regarding our submission and to respond to any further questions and comments you may have.

Sincerely,

Barbara Dritschel and Luisa Bergunde
